# Mind the gap: an intervention to support caregivers with a new autism spectrum disorder diagnosis is feasible and acceptable

**DOI:** 10.1186/s40814-020-00662-6

**Published:** 2020-09-07

**Authors:** Suzannah Iadarola, Melanie Pellecchia, Aubyn Stahmer, Hyon Soo Lee, Lindsay Hauptman, Elizabeth McGhee Hassrick, Samantha Crabbe, Sarah Vejnoska, Elizabeth Morgan, Heather Nuske, Paul Luelmo, Chris Friedman, Connie Kasari, Amanda Gulsrud, David Mandell, Tristram Smith

**Affiliations:** 1 University of Rochester Medical Center, 601 Elmwood Ave, Box 671, Rochester, NY 14642 USA; 2 University of Pennsylvania, 3535 Market St, 3rd floor, Philadelphia, PA 19104 USA; 3 University of California, Davis, 2825 50th St, Sacramento, CA 95817 USA; 4 University of California, Los Angeles, UCLA Semel Institute 68-268, Los Angeles, CA 90024 USA; 5 Drexel University, 3020 Market Street | Suite 560, Philadelphia, PA 19104 USA; 6 San Diego State University, 5500 Campanile Dr, San Diego, CA 92182 USA

**Keywords:** Autism spectrum disorder, service access, disparities, caregiver education

## Abstract

**Introduction:**

Children with autism spectrum disorder (ASD) benefit when their caregivers can effectively advocate for appropriate services. Barriers to caregiver engagement such as provider mistrust, cultural differences, stigma, and lack of knowledge can interfere with timely service access. We describe Mind the Gap (MTG), an intervention that provides education about ASD, service navigation, and other topics relevant to families whose children have a new ASD diagnosis. MTG was developed via community partnerships and is explicitly structured to reduce engagement barriers (e.g., through peer matching, meeting flexibility, culturally-informed practices). We also present on the results of a pilot of MTG, conducted in preparation for a randomized controlled trial.

**Methods:**

MTG was evaluated using mixed methods that included qualitative analysis and pre/post-test without concurrent comparison group. Participants (n=9) were primary caregivers of children (ages 2-7 years) with a recent ASD diagnosis and whose annual income was at or below 185% of the federal poverty level. In order to facilitate trust and relationship building, peer coaches delivered MTG. The coaches were parents of children with ASD who we trained to deliver the intervention. MTG consisted of up to 12 meetings between coaches and caregivers over the course of 18 weeks. Coaches delivered the intervention in homes and other community locations. Coaches shared information about various “modules,” which were topics identified as important for families with a new ASD diagnosis. Coaches worked with families to answer questions, set weekly goals, assess progress, and offer guidance. For the pilot, we focused on three primary outcomes: feasibility, engagement, and satisfaction. Feasibility was measured via enrollment and retention data, as well as coach fidelity (i.e., implementation of MTG procedures). Engagement was measured via number of sessions attended and percentage completion of the selected outcome measures. For completers (n=7), satisfaction was measured via a questionnaire (completed by caregivers) and open-ended interviews (completed by caregivers and coaches).

**Results:**

We enrolled 56% of referred caregivers and 100% of eligible families. Retention was high (78%). Coaches could deliver the intervention with fidelity, completing, on average, 83% of program components. Engagement also was high; caregivers attended an average of 85% of total possible sessions and completed 100% of their measures. Caregivers indicated moderately high satisfaction with MTG. Qualitative data indicated that caregivers and coaches were positive about intervention content, and the coach-caregiver relationship was important. They also had suggestions for changes.

**Conclusion:**

Mind the Gap demonstrates evidence of feasibility, and data from the pilot suggest that it addresses intervention engagement barriers for a population that is under-represented in research. The results and suggestions from participants were used to inform a large-scale RCT, which is currently underway. Overall, MTG shows promise as an intervention that can be feasibly implemented with under-resourced and ethnic minority families of children with ASD

**Trial registration:**

This study is registered with ClinicalTrials.gov: NCT03711799.

## Introduction

### Family Engagement: Benefits and Barriers

Engaging children with autism spectrum disorder (ASD) in early intervention improves child and family outcomes. Early intervention can maximize long-term outcomes; it therefore is critical that parents learn about and access services as soon as possible [1, 2]. Because children with ASD often access services across a range of providers and service systems, their caregivers often have substantial care coordination responsibilities [3]. Many studies have highlighted the difficulties families face in navigating the complex service systems for ASD [4], leading to negative outcomes. For example, Brewer [5] found that mothers reduced their paid work to meet the demands of accessing ASD-related services. The author highlighted the disproportional effects this has on parents from low-income households.

Delays in service access are especially common among families from traditionally under-resourced groups. Children from racial or ethnic minority groups are less likely than white children, and children from low-income households are less likely than higher-income children to be diagnosed with ASD at an early age [6, 7]. They are also more likely to start services at an older age and receive fewer services [6, 8]. Parents who are struggling financially often report limited guidance on steps to accessing services, and have identified a number of structural barriers (e.g., work schedule or transportation) to meeting their child’s needs [9, 10]. Many parents feel overwhelmed with managing their child’s support needs in the context of financial instability, stigma surrounding ASD, and isolation [11, 12]. Perhaps as a result of challenges in navigating service systems, parents of children with ASD often experience more stress and mental health problems than parents of children with other disabilities or typically developing children [13–15]. Parents of children with ASD also report that they do not understand the service options available to their child [10]. This is an important consideration, as parents’ service knowledge appears to be more important than socioeconomic status per se in predicting their children’s service use [9].

Another factor that may limit service engagement for low-resource families is their distrust of the medical system [16], based on negative experiences or mismatched cultural beliefs. Distrust of the service system is exacerbated by poor communication about diagnosis and treatment, inadequate access to treatment, and limited involvement of parents in decision-making about services [17–19]. For example, parents have reported that their children’s needs can become secondary to the battle over the cost of intervention and provider preferences for certain interventions [18]. Further, interventions for children with autism often are not culturally sensitive in a way that meets the needs of parents of color or under-resourced families [20, 21]. This cultural mismatch can result in underuse of services and low treatment adherence [21]. Therefore, the establishment of collaborative, supportive programming between parent and therapists/educators who are culturally competent and able to provide a variety of resources to economically diverse families is of utmost importance [10, 22].

### Addressing Engagement Barriers

Interventions designed to increase caregiver’s knowledge and advocacy have been successful for parents of children with ASD both as standalone interventions [23] and as components of more broad parent training [24]. One culturally inclusive parent intervention model for children of color involves using peer-mediated models, such as the Promotora de Salud Model. In peer-mediated approaches, individuals – such as lay community health workers – deliver interventions for parents that are culturally relevant and based upon shared experience [25]. Rather than representing a single *type of intervention*, peer-mediated intervention is an *approach* to delivering evidence-based information and services. It has been used to support Latinx families of children with ASD, with promotoras meeting with parents weekly in their homes. Preliminary evaluations suggest that this model is effective in increasing parent knowledge and access to community resources [26]. Further, the use of peer coaches to deliver interventions to parents has been identified as a critical element in increasing parent engagement in their child’s treatment [27]. These findings suggest the importance and promise of parent-centered interventions that are focused on service access and delivered by trusted peers with similar life experiences [10].

### Community Partner Input

Interventions that include partnership with key community stakeholders can result in more positive outcomes and more successful implementation than those that do not involve community engagement [28]. We developed the intervention described in this pilot study using an iterative community-partnered approach that incorporated stakeholder feedback and an in-depth exploration of the barriers to treatment engagement for under-resourced and minority caregivers of young children with ASD. Consistent with previous literature, caregivers reported system-level concerns (e.g., confusion around navigating service systems and a desire for greater support) and barriers related to cultural identity (e.g., community stigma, experiences with provider discrimination, limited language accessibility). They also wanted connection and guidance from others with similar life experiences [10], similar to the findings described in studies of peer-mediated models. These findings guided the development of our intervention – Mind the Gap – designed to explicitly address barriers to treatment engagement for under-resourced and minority caregivers of young children with ASD. In this study, we used a peer-mediated model to deliver Mind the Gap, which was a packaged intervention based on evidence-based information.

### The Present Study

Mind the Gap is a flexible, caregiver-focused intervention for families of young children with ASD. It addresses issues salient to caregivers (e.g., social support, system navigation, ASD knowledge, stress management) and children (e.g., challenging behavior, communication, service access) to support families in quickly accessing community services. Consistent with models using peer coaches with shared experience, our coaches were other parents of children with ASD, who are likely to engender trust with parents of newly diagnosed children based upon shared experience. The goal of Mind the Gap is to engage caregivers of young children with ASD to increase service use. We developed Mind the Gap using principles from the RE-AIM framework (*Reach, Efficacy, Adoption, Implementation, and Maintenance*) [29]*,* which aims to close the research-to-practice gap by developing interventions with both the participants and settings represented at each stage, and planning for and gathering data on implementation from the outset [30].

In preparation for a large-scale evaluation, we conducted a pilot study (n=9); our aims were to evaluate: 1) the feasibility of Mind the Gap delivery; 2) preliminary caregiver engagement outcomes; and 3) the feasibility of collecting outcomes data about caregivers. These aims are consistent with the Reach, Implementation, and Adoption phases of the RE-AIM framework. Data from this pilot supported a randomized controlled trial of Mind the Gap and informed intervention refinements for the full trial. An RCT designed to provide information on Efficacy, Implementation, and Maintenance is underway.

### Method

#### Design and Setting

We used a mixed-methods approach that included qualitative analysis and a pre/post-test study design without concurrent comparison group. We recruited participants from four sites: Los Angeles, CA; Sacramento, CA; Philadelphia, PA; and Rochester, NY. These sites comprise the Autism Intervention Research Network on Behavioral Health (AIR-B), a federally funded research network dedicated to improving outcomes for children with ASD and their families who experience income-based disparities. We therefore engaged with communities that included high rates of families living at or near the poverty line. Local Institutional Review Boards at each participating site approved the study procedures, with UCLA as the IRB of record (IRB# 17-000029). Although no changes to the pilot methodology were made during the trial, we tracked opportunities for refining the methods for the RCT phase. The length of the pilot was delineated as one year, to allow for completion of all intervention and assessment prior to the initiation of the RCT.

#### Recruitment and Participants

##### Caregivers

Participants included primary caregivers of children between 2 and 7 years of age that received a diagnosis of autism spectrum disorder within the last year (n=9). From April 2016 through July 2016, each site attempted to enroll 2 or 3 families; this sample size allowed for sufficient piloting of the intervention methods to identify needed modifications for the RCT. Inclusion criteria were: 1) the child could not be receiving any autism-specific services (e.g., high-intensity applied behavior analysis or special instruction,); 2) the family’s annual household income had to be at or below 185% of the federal poverty level; 3) the caregiver had to speak English or Spanish; and 4) the caregiver was willing to participate in the intervention for 12 weeks. Income criteria were set based upon the study aims of evaluating an intervention focused on reducing engagement barriers for under-represented families. There were no exclusion criteria regarding the child’s intellectual functioning, autism severity, communication skills or comorbid conditions. Caregivers could not participate if the child was in an out-of-home placement.

Sites engaged local community partners to identify viable streams of recruitment and create a system for processing referrals. Referrals came from local pediatrician offices, early intervention agencies, developmental behavioral pediatric clinics, local community organizations, family resource centers, and school staff. Caregivers could self-refer from posted recruitment flyers and social media. Research staff contacted interested families to provide more information and assess for eligibility.

##### Peer Coaches

Peer coaches (n=12) were recruited through partnerships with community organizations for participation in this pilot and the larger study. Each site recruited more peer coaches than needed to provide sufficient opportunities for cultural matching with participants. Sites worked with their local community partners to market the opportunity to parent and advocacy groups. Other recruitment methods included social media ads, and recruiting through providers from community clinics. Peer coach eligibility included: 1) having a child age 6 years or older with a diagnosis of ASD provided at least 3 years prior; 2) previous experience working with other parents and the service system in their area; 3) fluency in English or Spanish. Twelve parents participated as peer coaches. The coaches were primarily female with an average of 3 years previous experience working with families.

#### Mind the Gap Intervention

##### Development and Structure

We developed Mind the Gap through an iterative collaborative process with community stakeholders. A modular approach provides opportunities to individualize the intervention based on each family’s needs. Relevant topics were identified based upon data from focus groups [10], community partnership input, and from previous research. This study included seven modules (Table 1), each of which consisted of short informational videos, narrated PowerPoint presentations, infographics and other information sheets, and topic-related worksheets. In addition to didactic content, each module included engagement activities and goal-oriented tasks for the parent that the coach and parent identified together. The modules, materials, and videos were translated into Spanish.

**Table 1:** Mind the Gap Intervention Modules

Module	Topic
What is ASD?	Provides parents basic information about ASD and its characteristics.
Navigating the System	Information about how to access school-based, and healthcare services.
Stigma	Addresses cultural barriers that parents from under-resourced backgrounds may face.
Challenging Behavior	Helps parents learn basic techniques on preventing and de-escalating challenging behavior.
Anxiety and Acceptance	Provides parents with information about mental health needs after receiving a child’s diagnosis.
Healthy Lifestyle	Provides parents with information about self-care (e.g., sleep and exercise).
Working with Providers	Information about communication, and organization strategies for effectively working with services providers and other professionals.

Each caregiver was matched with one peer coach, who worked with the caregiver throughout the study. When possible, we matched caregivers and coaches on culture and language, with the goals of enhancing their ability to develop rapport and maximizing the likelihood of shared experiences. Prior to starting the intervention, peer coaches participated in an intensive, 12-hour training on: 1) components of the intervention; 2) data collection procedures; 3) caregiver engagement strategies; 4) community research ethics; and 5) ensuring safety and boundaries with research participants.

##### Intervention delivery

The intervention consisted of weekly meetings between peer coaches and caregivers; this phase lasted for 12 sessions, completed in up to 18 weeks to accommodate missed or cancelled meetings. Meetings could occur: 1) in-person, at the preferred location identified by the caregiver (e.g., home, library, community center), 2) via secure Zoom teleconferencing; or 3) over the phone. An initial in-person meeting between the caregiver and member of the research team included: a) provision of consent; b) confirmation of community diagnosis; c) completion of parent questionnaires; d) optional enrollment in Parent Square (on on-line, secure communication system); e) review of the Mind the Gap materials binder; and f) provision of initial information about the peer coach. *Intake meetings (Session 1)* took place with the peer coach in a location convenient for the family. During the meeting, the peer coaches followed a semi-structured intake interview script to establish rapport with the family, learn about goals for their child and family related to ASD, and identify the family’s basic needs. In subsequent meetings *(Sessions 2-12),* caregivers worked with their peer coaches to review relevant content and engage in an ongoing goal-setting process. Sessions occurred approximately every week by phone or in person, with the goal of at least one in-person meeting per month. Coaches followed a general session structure: a) reviewing progress since the previous session; b) assisting the caregiver in selecting a new topic to review; c) providing relevant information on the chosen topic (e.g., watching module videos, reviewing handouts, completing worksheets, providing resources); d) setting weekly next steps and documenting them on the family’s handout; and e) setting the data and time of the next session. Family needs and preferences guided topic selection. Coaches and caregivers collaboratively reviewed progress each week, with coaches providing additional assistance to overcome barriers. While engaged in active coaching, peer coaches participated in regular supervision meetings with study staff, who in turn were supervised by a licensed psychologist. Meetings involved a combination of didactics on identified topics (e.g., caregiver engagement, cross-cultural communication), case presentations, and group troubleshooting. If needed, the research team assisted the peer coach by providing supervision, consultation, or access to additional resources.

During intervention, peer coach/participant dyads could communicate via brief phone check-ins or Parent Square. Parent Square is an application available in English and Spanish that participants could use to communicate about the intervention and share information and resources via direct messages, posts, creating events, etc. Parents and peer mentors received training in how to use the app. Three sites used Parent Square, as the Rochester IRB did not approve its use. Coaches were encouraged to schedule meetings and share information through parent square, and to check the app for messages at least weekly.

### Primary (Feasibility) Outcomes

Feasibility outcomes were fourfold. First, we collected data on *enrollment and retention (i.e.,* ***Reach****)*; enrollment was defined as the percentage of participants who were screened and determined eligible and who consented to participate. Retention was defined as the percentage of participants who completed intervention and data collection at exit. A recent systematic review of parent engagement in child-focused interventions [27] for under-resourced families reported mean retention of 74%; we therefore aimed for over 80% retention.

Second, we tracked *intervention fidelity (i.e.,* ***Implementation****)*. The fidelity of peer coach implementation was measured through audio-recording and coding 25% of phone and in person sessions, as determined by an independent statistician who selected sessions using a random number generator. Following recording, a member of the research team coded the audio for fidelity using the fidelity of implementation checklist. Based on fidelity conventions, we aimed for a benchmark of 80% fidelity.

Third, we collected participant *engagement data*; this was a critical dimension of the pilot as these data directly provide information regarding how well Mind the Gap addressed common obstacles identified in the literature, given that a major development aim in Mind the Gap was reducing barriers to treatment engagement. We measured engagement in two ways: 1) number of sessions attended and 2) digital engagement data on communication via Parent Square. User data was extracted from the app using R programming. Count data relating to app user activities, including posting, private message, comments and resource uploading activity, were calculated for parents and peer mentors. This was an exploratory measure, given that there is limited research on digital engagement in these populations for child-focused interventions.

Fourth, we assessed *caregiver and coach satisfaction (i.e.,* ***Adoption****)* with MTG, using mixed-methods. Although not a pure measure of adoption, coach satisfaction was used as a proxy in this pilot, as provider (i.e., coach) satisfaction with an intervention would likely increase its uptake. Caregivers rated their satisfaction with the intervention on an 18-item *Caregiver Satisfaction Survey*, using a 5-point Likert scale to identify their degree of agreement with various statements (higher scores indicate more positive perceptions). Items asked about structure and content of Mind the Gap (e.g., “The topics we met covered my needs,” “The number of sessions seemed about right”). We aimed for families to indicate that they were mostly satisfied or better (i.e., average rating of 3.5 or above). To supplement quantitative data, we collected qualitative data via a *semi-structured interview on satisfaction* administered to caregivers and coaches by members of the research team. To obtain general impressions of the program, we asked open-ended questions about satisfaction with the intervention, the success of the relationship with the peer coach, and possible improvements to Mind the Gap. The data were then coded and summarized into overall feedback on caregiver and coach satisfaction.

### Secondary (Measure Completion) Outcomes

Participants completed self-report measures based on our theorized mechanisms of change. In the context of the pilot –and given the small sample size – pre/post comparisons of the data is inappropriate, given limited power. Instead, secondary outcomes focused on *completion of outcome measures*. Specifically, documenting the ability to collect complete data provides additional support for the feasibility of using these measures in a larger trial. We also present the mean scores on these measures at baseline and exit, both to characterize this pilot sample and to provide context to the interpretation of the findings.

### Caregiver Agency Questionnaire

The Caregiver Agency Questionnaire is adapted from Kuhn and Carter [31], and is a 10-item survey about how often the parent engaged in certain activities related to promoting child development. It yields a score range from 10 to 50, with higher scores being indicative of higher agency. While not yet a validated measure, an initial evaluation of caregiver agency has been conducted in parents of children with ASD [31].

### Maternal Autism Knowledge Questionnaire

This 10 item true/false questionnaire measures knowledge of facts about autism in the areas of diagnosis, symptoms, treatments and interventions, and etiology. It was adapted from a longer version, with permission of the survey author [31]. The percent of correctly answered questions constitutes the autism knowledge score.

### Caregiver Stigma Scale

This is an 11-item scale adapted from an unpublished measure [32] that assesses the degree of stigma that caregivers have about receiving professional services or treatment for their child from a mental health or developmental specialist (e.g. developmental pediatrician, psychologist, psychiatrist). It yields a score from 11 to 55, with higher scores indicative of higher stigma.

### Family Empowerment Scale (FES)

This is a 34-item measure [33] that measures empowerment in families with children who have emotional, behavioral, or developmental disorders. The FES has three subscales, Family, Service System and, Social Politics. Higher scores are indicative of higher family empowerment.

### Data Collection Procedures

Given the study aims, primary outcomes were measured weekly, and secondary outcomes were measured at baseline and post-treatment. The schedule of measures was organized around data collection for both caregiver and peer coach participants (see Table 2).

**Table 2:** Schedule of Measures

	Baseline	Within-Intervention	Post-Treatment
Parent-Completed Measures			
Demographics	X		
Family Empowerment Scale	X		X
Caregiver Agency Questionnaire	X		X
Caregiver Autism Knowledge Questionnaire	X		X
Caregiver Stigma Scale	X		X
Satisfaction Survey / Interview			X
Coach-Completed Measures			
Demographics	X		
Feasibility Survey			X
Research Team-Completed Measures			
Fidelity of Peer-Coach Implementation		X	
Access of Online Materials (ParentSquare)		X	X
Parent Attendance		X	X

### Data Collection Procedures for Pre/Post Measures

Caregiver participants completed baseline measures immediately following consent. Post-treatment data collection occurred within two weeks of the final session (i.e., session 12); this time point included completion of all parent questionnaires from baseline, with the addition of a parent Satisfaction Survey. Peer coach measures included the demographics questionnaire (baseline) and the feasibility interview (post-treatment).

### Data Collection Procedures for Measures used within the Intervention

Peer coaches completed engagement data (i.e., primary outcome data) weekly based upon parent attendance at meetings, topics covered, and goals completed. To ensure consistent implementation of Mind the Gap procedures, treatment fidelity data (i.e., ratings of peer coach implementation) were collected for 25% of sessions, which were randomly selected and recorded during the intervention phase. Recordings were rated against a checklist of procedures that were required for each visit. Each session fidelity was calculated as a percentage of number of procedures completed by the coach divided by the total number of procedures. Total fidelity is expressed as an average of all coaches’ session fidelity across sites.

### Data Analysis

Descriptive statistics for both primary and secondary measures are presented below. For the secondary outcome questionnaires, participant baseline characteristics are represented by the mean score on each questionnaire, as well as rates of completion on each measure. Given the small sample size, comparative statistics were not conducted, as we would be significantly underpowered to detect changes.

## Results

### Participant Demographics

Table 3 shows demographics for caregivers, their children, and peer coaches. Caregivers were largely female and Hispanic or Latinx; demographics were fairly equally distributed across education and income. A third of caregivers spoke Spanish and received the intervention in Spanish. The mean age of the children was 2.5 years, and most were male. Five caregivers were married or living with a partner and the others were either separated (22%), divorced (11%), or single (11%). All caregivers had an average income of less than $50,000 a year and fell below the federal poverty level standards based on the ratio of income to the number of people in the household.

**Table 3:** Participant demographics

		Caregivers	Peer Coaches	Children
Age: mean (SD)	Years	30.67 (6.4)	43.92 (4.7)	2.7 (1.0)
Gender: n (%)	Female	8 (89%)	11 (92%)	1 (11%)
	Male	1 (11%)	0 (0%)	8 (89%)
	Not reported	0 (0%)	1 (8%)	0 (0%)
Ethnicity: n (%)	Not Hispanic or Latino	3 (33%)	4 (33%)	1 (11%)
	Hispanic or Latino	6 (67%)	6 (50%)	8 (89%)
	Prefer not to Answer	0 (0%)	2 (17%)	0 (0%)
Race: n (%)	African American/Black	0 (0%)	3 (25%)	1 (11%)
	Caucasian/White	2 (22%)	6 (50%)	2 (22%)
	Prefer not to Answer	2 (22%)	0 (0%)	2 (22%)
	Asian American/Pacific Islander	0 (0%)	0 (0%)	0 (0%)
	Multiple	2 (22%)	2 (17%)	1 (11%)
	Other	3 (33%)	1 (8%)	3 (33%)
Primary Language: n (%)	English	5 (56%)	N/A	N/A
	Spanish	3 (33%)	N/A	N/A
	Other	1 (11%)	N/A	N/A
Education: n (%)	Some High School	1 (11%)	1 (8%)	N/A
	High School/GED	3 (33%)	0 (0%)	N/A
	Some College	3 (33%)	0 (0%)	N/A
	4-Year College Degree	2 (22%)	8 (67%)	N/A
	Master’s Degree or Higher	0 (0%)	2 (17%)	N/A
	Not reported	0 (0%)	1 (8%)	N/A
Income: n (%)	$9,000 or less	2 (22%)	N/A	N/A
	$10,000-19,999	2 (22%)	N/A	N/A
	$20,000-29,999	1 (50%)	N/A	N/A
	$30,000-39,999	2 (22%)	N/A	N/A
	$40,000-49,000	2 (22%)	N/A	N/A

### Feasibility outcomes

*Enrollment and Retention (i.e.,* ***Reach****)*.

**Fig. 1:**
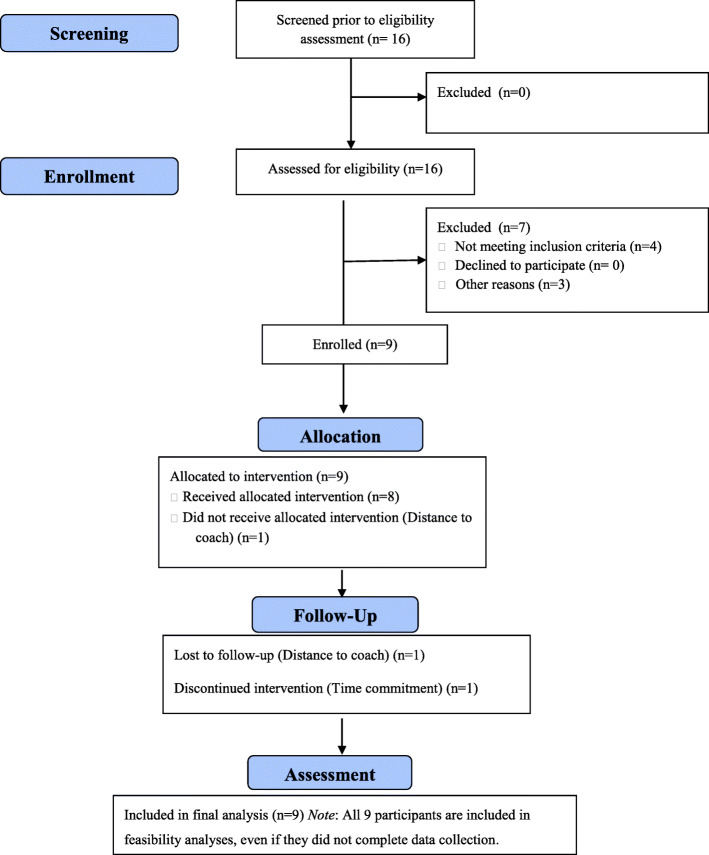
CONSORT Diagram

Referrals to Mind the Gap came primarily from community diagnostic clinics and state-funded resource centers. Out of 16 total screenings, nine caregivers were eligible and subsequently recruited and enrolled across four sites (UCLA: 3, Penn: 2, UC Davis: 2 and University of Rochester: 2) for the pilot study (i.e., 56% enrollment of total referrals and 100% enrollment of eligible families; see Fig. 1).

There was good retention, with 7 out of 9, or 78% of enrolled parents completing the twelve weeks of the intervention. One family withdrew from the study following the intake session, due to the time commitment. The assigned coach to the other family had difficulty reaching them for coaching during the intervention phase (i.e., following intake), and the family was eventually lost to follow-up.

*Coach Intervention Fidelity (i.e.,* ***Implementation****)*.

As rated by research staff, coach fidelity averaged 83% across sites, with individual ratings ranging from 66% to 100%.

*Caregiver and coach satisfaction (i.e.,* ***Adoption****)*.

Data from the Parent Satisfaction Questionnaire indicated moderate satisfaction with the Mind the Gap program (i.e., an average of 3.9 points per item out of a possible 4.5 points), with scores ranging from 2.2 to 4.5.

Qualitative interviews with coaches and parents indicated generally positive views toward most aspects of the intervention. Coaches reported that the training was helpful and that they benefitted from the support provided by the research team. Coaches also described the intervention technology as useful for sharing information and communicating with families. All coaches were greatly satisfied with the intervention content, including accessibility and helpfulness of the materials and modules, diversity and family centeredness of the topics, and the usefulness of the video modules. Both groups indicated a preference for a combination of in-person and phone meetings.

Parents and coaches described supportive relationships with each other. All parents mentioned that they were very happy with their coach, appreciated the relationship, and felt a sense of mutual understanding. Parents overwhelmingly viewed the coach as the most important and helpful aspect of the intervention. They noted, however, that is would be very important that caregivers and coach be matched on personality, and that matches should be arranged based on location, comfort with technology, and communication style. Other constructive feedback from coaches included that the data forms were cumbersome and overwhelming, with suggestions for improving their ease of use such as combining some of the forms and making them shorter. Parents suggested increasing the length of the intervention to allow for a more extended intervention period and increasing the time between sessions from weekly to twice monthly.

### Parent Engagement

#### Attendance

On average, parents attended ten of 12 sessions (85%). Most participants attended 12 sessions, but one family only completed four sessions. See Table 4 for a full description.

**Table 4:** Caregiver attendance at coaching sessions

	Number of Sessions Attended	Percent of Sessions Attended
Family 1	11	92
Family 2	12	100
Family 3	4	33
Family 4	12	100
Family 5	12	100
Family 6	12	100
Family 7	8	67
Family 8	1*	8
Mean (SD)**	10.1 (3.1)	84.5 (25.7)

* Participant withdrew following intake due to time constraints

** Excluding withdrawals

#### Digital Engagement

**Fig. 2:**
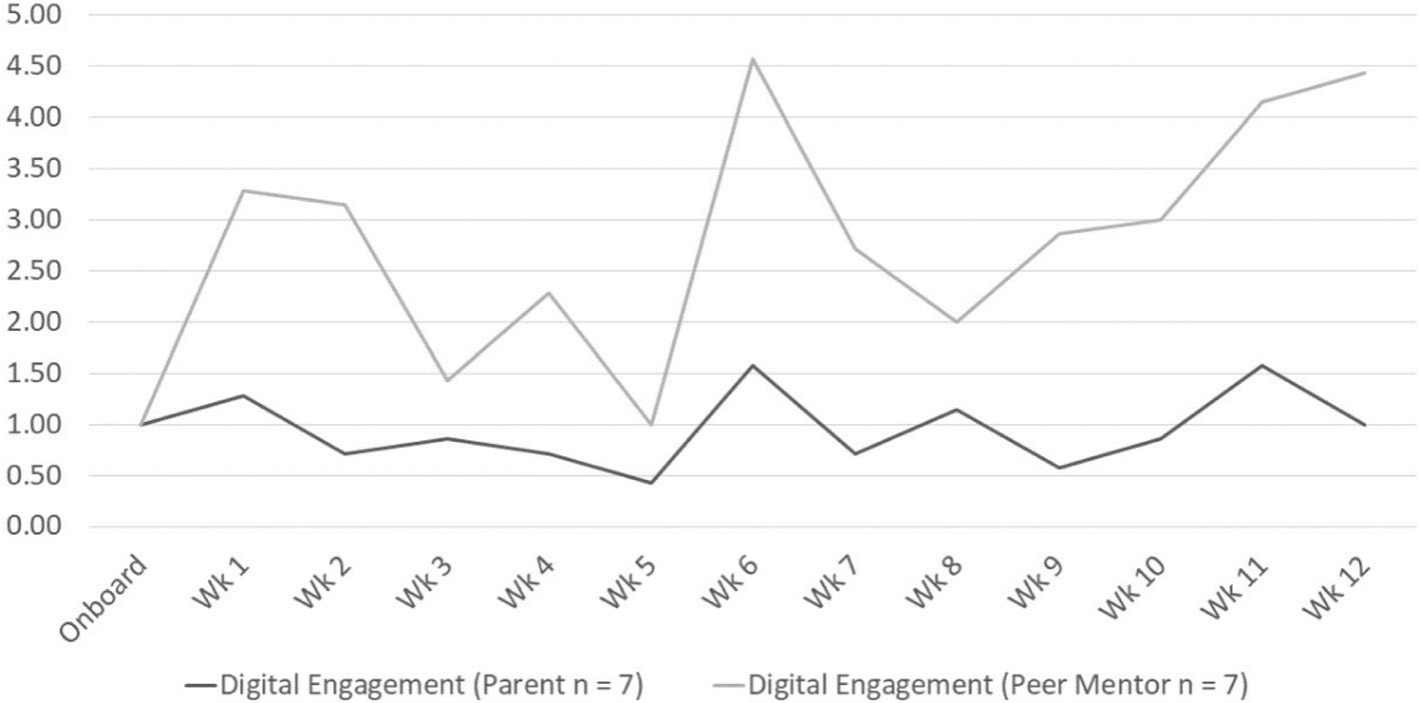
Within-Treatment Digital Engagement

All parent and peer coach pairs successfully started Parent Square accounts (Fig. 2). Most participants used the app to communicate during the intervention (parents =71%, peer mentors = 86%) and follow up period (parents =71%, peer mentors = 71%). Higher digital engagement occurred for both parents and peer coaches during the treatment period, with higher peaks in week 6 and 12 for peer mentors and similar lower peaks for parents in weeks 6, 12 and 16. Declining digital engagement occurred for both parents and peer coaches throughout the follow up period, with no participation after week 30 from either parents or peer coaches.

#### Completion of Measures

All parents completed all measures at baseline (Table 5). All parents who finished Mind the Gap also completed these measures again post-intervention. All but one participant completed the satisfaction measure at exit.

**Table 5:** Measure completion and baseline scores

	Baseline			Exit		
	Mean (SD)	Range	Percent Completed	Mean (SD)	Range	Percent Completed*
Caregiver Agency	40.6 (6.7)	29-50	100 (9/9)	40.4 (5.7)	30-48	100 (7/7)
ASD Knowledge	0.73 (0.1)	0.6-1.0	100 (9/9)	0.8 (0.1)	0.6-1.0	100 (7/7)
Stigma	48.6 (6.5)	39-55	100 (9/9)	50.3 (3.8)	43-55	100 (7/7)
Family Empowerment Family Subscale	48.7 (3.9)	41-53		50.4 (4.7)	45-56	
Family Empowerment Service Subscale	48.7 (9.63)	31-60		51.2 (5.2)	45-60	
Family Empowerment Involvement Subscale	33.8 (10.73)	20-54		32 (6.8)	25-40	
Family Empowerment Total	131 (19.8)	101-162	100 (9/9)	134.3 (15.8)	115-160	100 (7/7)
Satisfaction				3.85 (.84)	2.2 – 4.5	75 (6/8)

*Measured for those who completed intervention only

### Parent Report Measures

See Table 5 for a summary of the baseline and exit descriptive data.

#### Caregiver Agency Questionnaire

The mean baseline score on the Caregiver Agency Questionnaire was 40.6, indicating high agency prior to intervention. Although the adaptation of the measure used in this study has not been normed, the baseline findings are similar to data obtained on the original Maternal Agency Questionnaire, which found a mean rating of 32 (out of a possible range of 10-50) among mothers of children with ASD [31]. Following intervention, the mean score reported was 40.4, with a range of 30-48.

#### Autism Knowledge Questionnaire

At baseline, caregivers answered an average of 73% of questions correctly on the Autism Knowledge Questionnaire, which was lower than the 91% reported by the original developers of the measures [31]. At exit, caregivers answered an average of 80% of questions correctly.

#### Caregiver Stigma Scale

Caregivers reported relatively high stigma at baseline (*M*=48.6), and stigma was similar at exit (*M*=50.3).

#### Family Empowerment Scale

At baseline, scores on the Family Empowerment Scale fell at the moderate levels across subscales, with the Family (*M*=48.7) and Service (*M*=48.7) subscales being somewhat higher than the Involvement subscale (*M*=33.8). Of note, the ranges on the Service subscale narrowed from baseline (31-60) to exit (45-60). The total baseline average of 131.1 (range 101-162) was comparable [33] or slightly lower [34] than those reported in other samples of parents of children with emotional disabilities involved in navigating the service system. Following intervention, caregivers reported a Total Score of 134.3 and a narrower range of scores (115-160).

## Discussion

We used an iterative, community-partnered approach to develop a modular, peer-led service navigation intervention, Mind the Gap, designed to increase service access for under-served parents of young children with ASD. In an effort to improve the intervention's ecological validity, stakeholder feedback guided the development of Mind the Gap to address barriers and capitalize on facilitators to service engagement [10]. In addition to community input, the intervention also drew from research showing improved parent engagement when a peer with similar life experiences delivers the intervention [26]. The goal of this pilot study was to provide preliminary evidence regarding Mind the Gap’s feasibility, acceptability, and efficacy, and to inform adaptations in preparation for a large multi-site randomized trial.

Users who find interventions acceptable are more likely to implement them successfully and sustain their use [35]. Mind the Gap demonstrated high acceptability from parents and peer coaches for content and delivery method. Parents also reported that they felt more empowered and effective in navigating their child’s treatment upon completing the intervention. Overwhelmingly, parents reported that the relationship with their peer coach was the most essential and helpful aspect of the intervention. In follow-up interviews, all parents described a supportive relationship with their peer coaches built on trust and mutual respect. like those receiving other peer coaching interventions [26, 36], parents who received Mind the Gap valued the support of the peer coach above other aspects of the intervention, though it is unclear if access to the materials without peer coaching would have been sufficient to improve family outcomes.

We assessed preliminary implementation outcomes for Mind the Gap using the guiding principles of the RE-AIM framework (*Reach, Efficacy/Effectiveness, Adoption, Implementation, and Maintenance*) [29]. Our preliminary findings indicate that Mind the Gap is feasible, and acceptable to both parents and peer coaches. A critical aspect of an intervention’s feasibility of implementation is its *Reach*, or the proportion of individuals who are willing to participate in an intervention [30]. The high rates of enrollment and retention of participants within this study, especially those from under-resourced and ethnic minority groups who are often not represented in research, indicate that Mind the Gap is likely to have high Reach for families and children who are vulnerable to poor long-term outcomes.

We also assessed Mind the Gap’s *Implementation,* or the extent to which the intervention was delivered as intended [30], by evaluating the peer coaches’ intervention fidelity. Peer coaches achieved acceptable intervention fidelity, indicating that trained community providers can implement Mind the Gap as intended. The fact that intervention fidelity was high is notable given that the interventionists were new to research and were not clinicians. The RE-AIM framework defines effectiveness as the impact of the intervention on important outcomes. Although the primary aims of this pilot study were to assess Mind the Gap’s feasibility and acceptability, we collected data regarding the intervention’s potential *effectiveness.* Modest improvements were noted in family empowerment and autism knowledge, while caregiver agency and stigma remained stable. Further, the narrowing of reported ranges (i.e., on Family Empowerment Total and Service subscale) at exit indicate that changes on these measures are reasonable following the intervention period, which supports their use in a full-scale trial. The small sample of this pilot study precluded meaningful analyses of these outcome data; however high rates of survey completion indicate the feasibility of collecting these outcome data within a larger randomized trial that lends itself to rigorous statistical analyses.

### Informing the RCT

The findings from this pilot study informed a large-scale multi-site randomized trial of Mind the Gap, which is currently underway. Parent, coach, and stakeholder feedback from the pilot informed modifications to the intervention for the randomized trial. First, we developed a more flexible approach to delivering the intervention that allowed for fewer required in-person visits and more options for phone-based sessions, in order to decrease the level of burden for coaches and parents. Similarly, we added video chat options for supervision meetings with the research team and parent-coach meetings. We extended the timeline for completion of sessions to reduce burden and allow for flexibility in session delivery. Second, parent and coach feedback informed changes to the intervention content, including adding additional modules to support families through life stressors outside of the scope of their child’s ASD services, such as accessing health insurance and addressing food insecurity, as these issues were prevalent among many of the participating families and were often at the forefront of families’ concerns. We now consider additional caregiver and coach characteristics in the matching process for the RCT. Specifically, we enhanced the coach interview process to obtain additional information that was used to develop a matching questionnaire that included new questions about preference for location of peer coach, preferred communication style, and the age/gender of the peer coach’s child. To ensure that peer coaches were sufficiently past the adjustment to diagnosis process for their own child, we raised the enrollment requirement for the age of the coach’s child to 9 years. Other changes to the intervention being implemented in the current randomized trial include: simplifying data forms, providing additional training to coaches, converting text-heavy information for parents into easily understood infographics, and translating the intervention into Korean to include an even more diverse sample of families in the study.

### Limitations

Several limitations are worth noting. First, the study design did not allow for a comparison group, which limits our ability to determine whether the preliminary efficacy outcomes noted were linked directly to intervention. Second, one of the four sites did not use ParentSquare, one of our engagement measures, due to local IRB constraints. Although we were able to measure attendance in all four sites as a measure of engagement, it would have been preferred to have the electronic measure of engagement across sites. Despite this, the high rates of attendance at all four sites, and overall high rates of engagement through the digital platform provide promise for Mind the Gap’s effectiveness in improving parent engagement. Finally, the high rates of engagement data we observed with Parent Square may have been influenced by the study team’s encouragement for and support of its use, through communication with the coaches. Caregivers may not replicate this level of engagement without ongoing reminders to use the app,

## Conclusion

There is a critical need for interventions that can be feasibly implemented and are effective in successfully engaging traditionally under-represented families of children with ASD in their child’s treatment. Mind the Gap shows promise as an intervention that can be feasibly implemented with under-resourced and ethnic minority families of children with ASD. The intervention was highly accepted by parents and was implemented with fidelity by lay peer coaches. The community-partnered development process, along with ongoing stakeholder feedback, led to the development of an intervention that is ecologically valid and appropriate for implementation within under-resourced community settings. A rigorous evaluation of Mind the Gap’s effectiveness at improving parent engagement and service access is currently in process, and a more robust evaluation of RE-AIM within this context will provide information on the intervention’s potential to be fully implemented.

## Data Availability

The datasets generated and/or analysed during the current study are not publicly available due to the full trial still being underway, but they are available from the corresponding author on reasonable request.
